# Supplementary material for: Characteristics of the vaginal microbiome in cross-border female sex workers in China: a case-control study
Source: PeerJ. 2019 Nov 29;7:e8131. doi: 10.7717/peerj.8131 (PMC6886492; doi:10.7717/peerj.8131)
Supplement: Supplemental Information 1 [file peerj-07-8131-s001.docx]

Supplement table 1. PERMANOVA analysis for potential variables on the microbiota community.

|  | *R*^2^ | *P* |
| --- | --- | --- |
| FSW vs Control | 0.321 | 0.001 |
| Vaginitis vs Non-Vaginitis | 0.032 | 0.060 |
| Pregnancy histrory* | 0.061 | 0.028 |
| Age* | 0.090 | 0.002 |

*These continuous variables were categorised as follow: pregnancy histrory, 0 vs 1 vs ≥2; age, ≤24 vs 25-29 vs ≥30 years old.
