# Supplementary material for: Characteristics of the vaginal microbiome in cross-border female sex workers in China: a case-control study
Source: PeerJ. 2019 Nov 29;7:e8131. doi: 10.7717/peerj.8131 (PMC6886492; doi:10.7717/peerj.8131)
Supplement: Supplemental Information 2 [file peerj-07-8131-s002.docx]

Supplement table 2. The alpha diversity of female sex workers and non-sex workers. Data were presented as median (interquartile range).

| Alpha diversity | Female Sex Workers  N=23 | Non-Sex Workers  N=37 | Z* | P | Adjust F^#^ | Adjust P |
| --- | --- | --- | --- | --- | --- | --- |
| ACE | 45.14（11.31） | 41.88（12.03） | -1.558 | 0.119 | 0.572 | 0.453 |
| Chao1 | 44.20（11.83） | 39.33（13.04） | -1.574 | 0.116 | 0.270 | 0.606 |
| Simpson | 0.602（0.471） | 0.784（0.478） | -1.133 | 0.257 | 0.044 | 0.834 |
| Shannon | 0.779（0.808） | 0.515（0.848） | -1.072 | 0.284 | 0.007 | 0.933 |

* Z values were caculated from Mann-Whitney Test

# F values were adjusted from age, the number of pregnancy history and vaginitis history by analysis of covariance (ANCOVA)
