# Supplementary material for: Characteristics of the vaginal microbiome in cross-border female sex workers in China: a case-control study
Source: PeerJ. 2019 Nov 29;7:e8131. doi: 10.7717/peerj.8131 (PMC6886492; doi:10.7717/peerj.8131)
Supplement: Supplemental Information 3 [file peerj-07-8131-s003.docx]

Supplement table 3. The distributions of vaginal micommunity state types (CSTs) between female sex workers and non-sex workers. Data were presented as frequencies (%)

| CST | FSW | FNSW | χ^2^ | P |
| --- | --- | --- | --- | --- |
| Lactobacillus Dominant （CST I, II, III） | 13（56.52） | 25（67.57） | 0.745 | 0.388 |
| High Diversity Dominant (CST IV) | 10（43.48） | 12（32.43） |  |  |
| Specific CSTs |  |  |  |  |
| CST I | 1（4.35） | 8（21.62） |  | 0.303* |
| CST II | 1（4.35） | 1（2.70） |  |  |
| CST III | 11（47.83） | 16（43.24） |  |  |
| CST IV | 10（43.48） | 12（32.43） |  |  |

* This is the result of Fisher exact test.
