# Supplementary figures and images for: Characteristics of the vaginal microbiome in cross-border female sex workers in China: a case-control study
Source: PeerJ. 2019 Nov 29;7:e8131. doi: 10.7717/peerj.8131 (PMC6886492; doi:10.7717/peerj.8131)

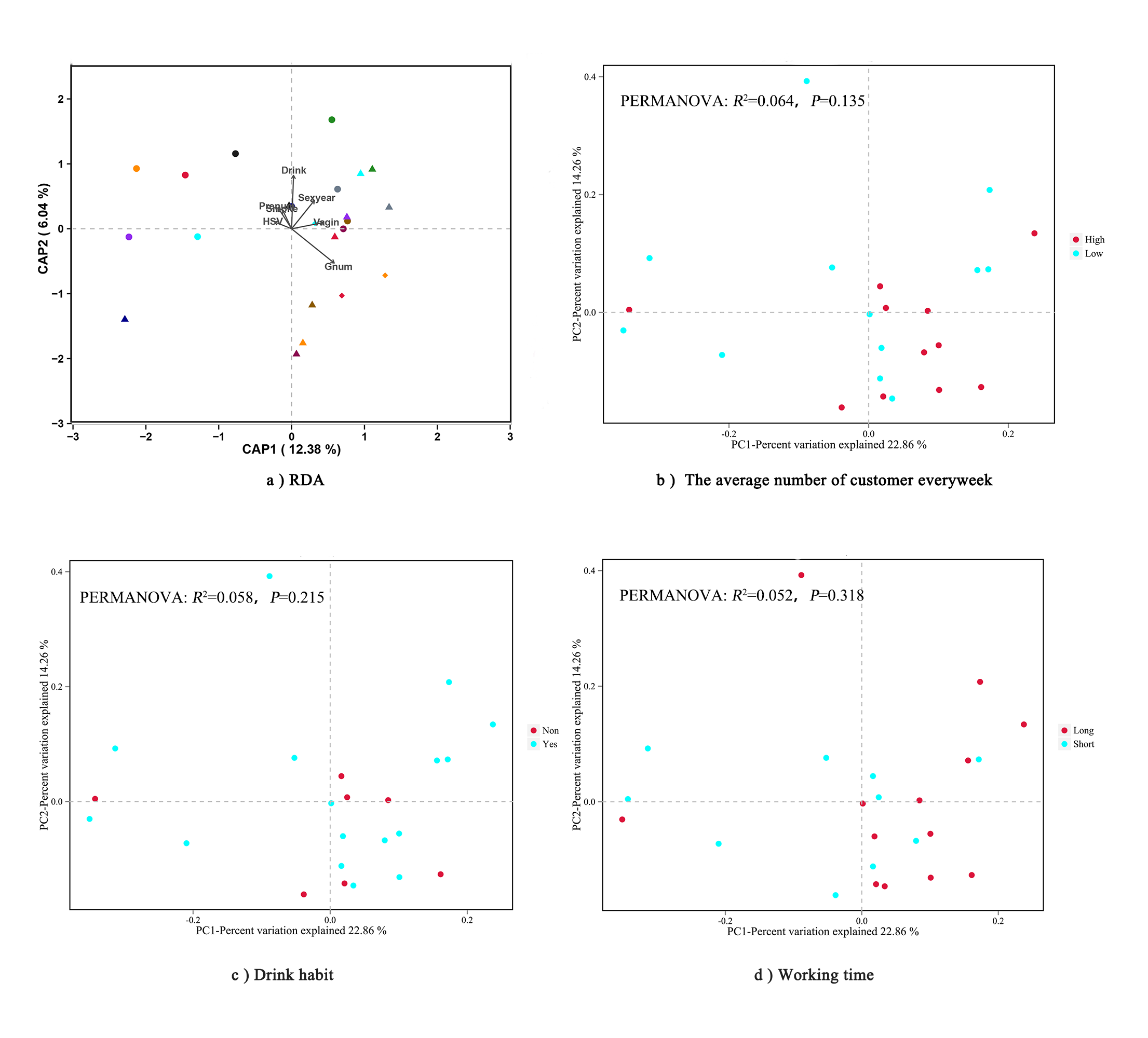

Supplement: Supplemental Information 4 [file peerj-07-8131-s004.png]
